# Hyperglycaemia in Pregnancy Is Less Frequent in Smokers: A French Observational Study of 15,801 Women

**DOI:** 10.3390/jcm13175149

**Published:** 2024-08-30

**Authors:** Emmanuel Cosson, Sopio Tatulashvili, Eric Vicaut, Lionel Carbillon, Hélène Bihan, Imen Rezgani, Sara Pinto, Meriem Sal, Mohamed Zerguine, Marion Fermaut, Jean-Jacques Portal, Jardena J. Puder, Amélie Benbara

**Affiliations:** 1 AP-HP, Avicenne Hospital, Sorbonne Paris Cité, Department of Endocrinology-Diabetology-Nutrition, CRNH-IdF, CINFO, Paris 13 University, 93000 Bobigny, France; sopio.tatulashvili@aphp.fr (S.T.); helene.bihan@aphp.fr (H.B.); imen.chelbi@aphp.fr (I.R.); sara.pinto@aphp.fr (S.P.); meriem.sal@aphp.fr (M.S.); mohamed.zerguine@aphp.fr (M.Z.); 2 INSERM, INRAE, CNAM, Center of Research in Epidemiology and StatisticS (CRESS), Nutritional Epidemiology Research Team (EREN), Université Sorbonne Paris Nord and Université Paris Cité, 93017 Bobigny, France; 3 AP-HP, Unité de Recherche Clinique St-Louis-Lariboisière, Université Denis Diderot, 75010 Paris, France; eric.vicaut@aphp.fr (E.V.); jean-jacques.portal@aphp.fr (J.-J.P.); 4 AP-HP, Jean Verdier Hospital, Sorbonne Paris Cité, Department of Obstetrics and Gynecology, Paris 13 University, 93140 Bondy, France; lionel.carbillon@aphp.fr (L.C.); marion.fermaut@aphp.fr (M.F.); amelie.benbara@aphp.fr (A.B.); 5 Obstetric Service, Department Woman-Mother-Child, Lausanne University Hospital, 1000 Lausanne, Switzerland; jardena.puder@chuv.ch

**Keywords:** cigarettes, diabetes in pregnancy, gestational diabetes mellitus, hyperglycaemia in pregnancy, oral glucose tolerance test, pregnancy outcomes, screening, smoking, tobacco

## Abstract

**Background:** We aimed to explore the still-debated association between smoking and hyperglycaemia in pregnancy (HIP). **Methods:** A multiethnic prospective study of 15,801 women who delivered at Jean Verdier University Hospital between 2012 and 2018. Of these, 13,943 (88.2%) were non-smokers, 624 (4.5%) former smokers, and 1234 (7.8%) current smokers. Universal HIP screening was proposed to the entire sample (IADPSG/WHO criteria). **Results:** A total of 13,958 women were screened for HIP. Uptake differed between non-smokers, former smokers, and current smokers (89.5%, 88.3%, and 75.7%, respectively, *p* < 0.0001). HIP prevalence in these groups was 19.9%, 15.4%, and 12.3%, respectively (*p* < 0.0001). After adjusting for age, body mass index, family history of diabetes, history of HIP, history of macrosomic baby, and ethnicity, current (odds ratio 0.790 [95% confidence interval 0.636–0.981], *p* < 0.05) but not former (1.017 [0.792–1.306]) smokers were less likely to have HIP than non-smokers. Furthermore, 1 h and 2 h oral plasma glucose test values were lower in current smokers than in non-smokers (*p* < 0.01). To exclude potential selection bias, we compared risk factors for HIP and HIP-related adverse pregnancy outcomes in current smokers according to HIP screening status. Compared with screened current smokers (n = 934), their unscreened counterparts (n = 300) were younger, less frequently employed, and more likely to be of non-European origin. Moreover, infant birthweight was lower in this group, and preterm deliveries and perinatal deaths were more likely (all *p* < 0.01). **Conclusions:** Smoking during pregnancy was independently associated with lower HIP prevalence. The low HIP screening rate in current smokers did not explain this finding.

## 1. Introduction

Hyperglycaemia in pregnancy (HIP) is associated with adverse pregnancy outcomes [1,2] and its prevalence is increasing worldwide [3,4]. Prevention is critical to ensure good health in mothers and their newborns [1,2]. According to the International Association of Diabetes Pregnancy Study Group (IADPSG), HIP covers two pathological conditions: gestational diabetes mellitus (GDM) and diabetes in pregnancy (DIP) [1]. DIP is defined as having test glucose values equal to or higher than the thresholds defining diabetes outside pregnancy. DIP diagnosis suggests undiagnosed type 2 diabetes before pregnancy [1,2].

Tobacco smoking outside pregnancy is still common worldwide [5]. In France, its prevalence is actually increasing [6]. Active smoking increases the risk of both pre-diabetes and diabetes [6], and these associations are dose-dependent [7]. Outside pregnancy, smoking also increases insulin resistance and has been identified as a modifiable risk factor for impaired insulin secretion, likely mediated by nicotine’s effect on beta-cell function [6].

Insulin resistance and insufficient insulin secretion are also determinants of HIP [1,2], and in theory, smoking during pregnancy encourages HIP [6]. Accordingly, smoking during pregnancy could be a modifiable risk factor for HIP. However, two recent meta-analyses did not find any significant association between current tobacco smoking and HIP [8,9]. This was also true for former smokers in one of the meta-analyses [8]. This may be due to confounding factors, such as younger age and lower body mass index (BMI) in smokers than non-smokers [6], as ageing and overweightness/obesity are risk factors for HIP [1,2,10]. Additionally, analyses in published studies may suffer from selection bias, as smokers generally tend to screen for diseases less than non-smokers [11,12,13].

In this context, using the IADPSG/World Health Organization (WHO) diagnostic criteria for HIP, we aimed to evaluate, in a large French dataset, whether smoking before and during pregnancy was associated with more prevalent HIP, independent of confounding factors, while taking into account potential recruitment bias.

## 2. Materials and Methods

### 2.1. Cohort Study

This observational prospective cohort study was conducted at Jean Verdier University Hospital in Bondy, a suburb of Paris, France. Analyses were based on data from the hospital’s routine electronic medical records of maternal and neonatal events at birth which occurred between January 2012 and December 2018 [10,14,15,16]. Data were analysed anonymously. Our database was declared to the French Data Protection Agency *(Commission Nationale de l’Informatique et des Libertés*, number 1704392v0).

### 2.2. HIP Screening and Care

We followed French recommendations for HIP screening, diagnostic criteria and care [2], and we recommended universal screening, given the high prevalence of risk factors in our hospital population. Screening is scheduled at the beginning of pregnancy; it is also performed after 24 weeks of gestation (WG) if the initial scheduled screening is not performed for some reason, or when the result of the initial screening is normal. Early screening at the beginning of pregnancy is based on the fasting plasma glucose (FPG) measurement. Women with an FPG level ≥ 5.1 mmol/L (92 mg/dL) are promptly provided care for HIP. Women not diagnosed early with HIP undergo an oral glucose tolerance test (OGTT) between 24 and 28 WG, where FPG and plasma glucose one (1h-PG) and two hours after OGTT (2h-PG) are measured.

We used the IADPSG/WHO [1] recommendations to diagnose HIP in accordance with French regulations [2]. Specifically, GDM is defined as an FPG level 5.1–6.9 mmol/L (92–125 mg/dL) and/or 1h-PG ≥ 10.0 mmol/L (180 mg/dL) and/or 2h-PG 8.5–11.0 mmol/L (153–199 mg/dL), while DIP is defined as an FPG ≥ 7.0 mmol/L (126 mg/dL) and/or a 2h-PG ≥ 11.1 mmol/L (200 mg/dL), both after 24 WG.

### 2.3. Selection Criteria for the Present Study Sample

The inclusion criteria for the women in the present study were as follows: delivery between January 2012 and December 2018 at Jean Verdier hospital, aged ≥18 years, no known diabetes before pregnancy, single-foetus pregnancy, no history of bariatric surgery, available smoking status, and finally, available HIP screening status (flow chart in Figure S1). Women who began smoking during pregnancy were excluded.

### 2.4. Data Collection

Smoking status was self-reported and categorized into three categories: women who were not smokers at the time of conception were classified as ‘non-smokers’; those who ceased smoking when they discovered they were pregnant were classified as ‘former smokers’, and those who continued smoking during their pregnancy were classified as ‘current smokers’. BMI was calculated according to self-reported weight before pregnancy and to height (measured by a professional) during pregnancy. Alcohol and drug consumption during pregnancy were self-reported. Ethnicity was also self-reported from one of six categories: European, North African, Sub-Saharan African, Indian-Pakistan-Sri Lankan, Caribbean, and Other.

### 2.5. Maternal and Neonatal Outcomes

We considered the following set of outcomes, using ‘maternal’ and ‘neonatal’ outcomes defined by the INSPIRED research group [10,14,15,16]. Maternal outcomes included gestational weight gain (GWG), planned and unplanned caesarean section, and hypertensive disorders. GWG was calculated as the weight measured before delivery minus the self-reported pre-pregnancy weight. Neonatal outcomes included birthweight, large-for-gestational-age (LGA) infant and small-for-gestational-age infant (defined as a birth weight greater than the 90th percentile and lower than the 10th percentile for a standard French population), gestational age at birth, and preterm delivery (any birth occurring after 22 WG + 6 days and before 37 WG), neonatal hypoglycaemia, perinatal death (death in the first 24 h of life or stillbirth), and, finally, any birth malformation. Definitions of these events are provided in previous publications [10,14,15,16].

### 2.6. Statistical Analyses

Baseline continuous variables were expressed as the mean ± standard deviation. Categorical variables were expressed as frequencies (percentages). No data replacement procedure was used for missing data.

Patient characteristics (Table 1, study sample) were compared according to smoking status with ANOVA for continuous variables, and the Chi-squared test or Fisher’s exact test for categorical variables. We performed a post hoc analysis to conduct inter-group comparisons using Dunnett’s alpha risk correction for multiplicity.

We also compared the rates of HIP according to smoking status (reference: non-smokers) using multivariable logistic regression analyses adjusted for the following usual confounders [1,2]: age, BMI, family history of diabetes, previous pregnancies with HIP, history of macrosomic child, and ethnicity (Table 2).

The smokers’ characteristics (Table 3) and adverse pregnancy outcomes (Table 4) were compared according to HIP screening uptake status. Finally, we also compared the rates of LGA infant and preterm delivery according to HIP screening status using multivariable logistic regression analyses adjusted for the parameters differing by screening status (i.e., age, employment at the beginning of pregnancy, parity, and ethnicity (Table 3)).

All tests were two-sided. Analyses were conducted using SAS 9.4 software (SAS Institute Inc., Cary, NC, USA).

## 3. Results

### 3.1. Study Population Characteristics

Among the 15,801 women who met the inclusion criteria (flow chart in Figure S1), 1843 (11.7%) were not screened for HIP. Of the 13,958 women who were screened, 2685 (19.2%) had HIP. Early diagnosed GDM, GDM, and DIP accounted for 4.8%, 13.3%, and 1.1% of the screened sub-population, respectively.

Table 1 describes the characteristics of the study population according to smoking status: non-smokers (n = 13,943, 88.2%), former smokers (n = 624, 4.0%), and current smokers (n = 1234, 7.8%). Compared with non-smokers (10.5%), current smokers were more likely not to screen for HIP (24.3%, odds ratio 2.73 [95% confidence interval 2.37–3.14]), while former smokers had a similar likelihood (11.7%: odds ratio 1.12 [95% confidence interval 0.88–1.44]).

**Table 1:** Characteristics of the study sample according to smoking status.

	Available Data	Non-Smokers	Former Smokers	Current Smokers	Total	*p*-Value
		n = 13,943	n = 624	n = 1234	n = 15,801	
Metabolic characteristics						
Age (years)	n = 15,801	30.5 ± 5.6	29.5 ± 5.6 *	28.7 ± 6.1 *	30.3 ± 5.6	<0.0001
Pre-pregnancy body mass index (kg/m^2^)	n = 15,132	25.1 ± 5.0	24.3 ± 5.2 *	23.6 ± 4.7 *	25.0 ± 5.0	<0.0001
Pre-pregnancy obesity	n = 15,132	2259 (16.9%)	90 (14.6%)	142 (12.1%) *	2491 (16.5%)	0.0001
Family history of diabetes	n = 15,801	3551 (25.5%)	192 (30.8%)	346 (28.0%) *	4089 (25.9%)	0.0025
Employment at beginning of pregnancy	n = 15,543	5069 (37.0%)	335 (54.0%) *	492 (40.5%) *	5896 (37.9%)	<0.0001
Parity	n = 15,801	2.18 ± 1.28	1.72 ± 1.05 *	2.08 ± 1.25 *	2.15 ± 1.27	<0.0001
Ethnicity	n = 15,777					<0.0001
Sub-Saharan African		3081 (22.1%)	58 (9.3%)	72 (5.9%)	3211 (20.4%)	
North African		4250 (30.5%)	87 (13.9%)	195 (15.9%)	4532 (28.7%)	
Caribbean		754 (5.4%)	63 (10.1%)	46 (3.7%)	863 (5.5%)	
European		3339 (24.0%)	351 (56.3%)	745 (60.6%)	4435 (28.1%)	
Indian–Pakistan–Sri Lankan		1485 (10.7%)	3 (0.5%)	2 (0.2%)	1490 (9.4%)	
Other		1014 (7.3%)	62 (9.9%)	170 (13.8%)	1246 (7.9%)	
Previous pregnancy history						
History of HIP	n = 15,801					<0.0001 §
First child/parity = 0		5098 (36.6%)	351 (56.3%)	510 (41.3%)	5959 (37.7%)	
No		8082 (58.0%)	260 (41.7%)	690 (55.9%)	9032 (57.2%)	
Yes		763 (5.5%)	13 (2.1%)	34 (2.8%) *	810 (5.1%)	
History of macrosomic child	n = 15,801					0.0131 §
First child/parity = 0		5098 (36.6%)	351 (56.3%)	510 (41.3%)	5959 (37.7%)	
No		8396 (60.2%)	260 (41.7%)	705 (57.1%)	9361 (59.2%)	
Yes		449 (3.2%)	13 (2.1%)	19 (1.5%) *	481 (3.0%)	
History of renal vascular disease in pregnancy	n = 15,801					0.1454 §
First pregnancy/parity = 0		3364 (24.1%)	206 (33.0%)	239 (19.4%)	3809 (24.1%)	
No		10,246 (73.5%)	409 (65.5%)	973 (78.8%)	11,628 (73.6%)	
Yes		333 (2.4%)	9 (1.4%)	22 (1.8%)	364 (2.3%)	
History of perinatal death	n = 15,801					0.4583 §
First pregnancy/parity = 0		3364 (24.1%)	206 (33.0%)	239 (19.4%)	3809 (24.1%)	
No		10,258 (73.6%)	409 (65.5%)	969 (78.5%)	11,636 (73.6%)	
Yes		321 (2.3%)	9 (1.4%)	26 (2.1%)	356 (2.3%)	
History of foetal growth restriction	n = 15,801					<0.0001 §
First pregnancy/parity = 0		3364 (24.1%)	206 (33.0%)	239 (19.4%)	3809 (24.1%)	
No		10,087 (72.3%)	404 (64.7%)	919 (74.5%)	11,410 (72.2%)	
Yes		492 (3.5%)	14 (2.2%)	76 (6.2%) *	582 (3.7%)	
Behaviours during pregnancy						
Alcohol consumption	n = 15,801	10 (0.1%)	0 (0.0%)	11 (0.9%) *	21 (0.1%)	<0.0001
Recreational substance consumption	n = 15,801	22 (0.2%)	14 (2.2%) *	62 (5.0%) *	98 (0.6%)	<0.0001
No HIP screening	n = 15,801	1470 (10.5%)	73 (11.7%) *	300 (24.3%) *	1843 (11.7%)	<0.0001
Glycaemic status during pregnancy	n = 13,958					<0.0001
Not diagnosed with HIP during screening		9988 (80.1%)	466 (84.6%)	819 (87.7%)	11,273 (80.8%)	
Early diagnosed gestational diabetes mellitus		621 (5.0%)	22 (4.0%)	30 (3.2%)	673 (4.8%)	
Gestational diabetes mellitus		1712 (13.7%)	62 (11.3%)	78 (8.4%)	1852 (13.3%)	
Diabetes in pregnancy		152 (1.2%)	1 (0.2%)	7 (0.7%)	160 (1.1%)	

Data are shown as n (%) or mean ± standard deviation. HIP: hyperglycaemia in pregnancy; OGTT: oral glucose tolerance test; WG: weeks of gestation. *: vs. No smoking, symbol inserted only if significant (*p* < 0.05) after Dunnett adjustment for multiplicity. §: Yes vs. No (no history possible if first child/parity = 0).

Age, BMI, family history of diabetes, employment, parity, ethnicity, and previous pregnancy history differed according to smoking status. Current smokers were more likely to drink alcohol than non-smokers (0.9% vs. 0.1%, respectively, odds ratio 12.5 [95% confidence interval 5.3–29.6]), and to consume recreational substances (5.0 vs. 0.2%, respectively, odds ratio 33.5 [95% confidence interval 20.5–54.6]) (Table 1).

### 3.2. HIP Prevalence According to Smoking Status

**Figure 1:**
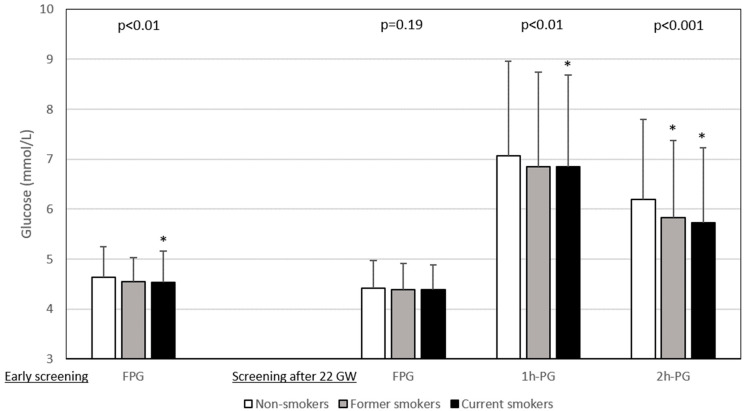
Plasma glucose values during hyperglycaemia in pregnancy screening according to smoking status. Data are shown as number ± deviation standard. Left panel: early screening (before 22 weeks of gestation (WG)), only fasting plasma glucose (FPG) value was measured (n = 5495). Right panel: after 22 WG, a 75 g oral glucose tolerance test (OGTT) was performed with measurement of FPG (n = 9588) and plasma glucose 1 h (1h-PG, n = 8716) and 2 h (2h-PG, n = 8879) after OGTT. Gestational age at early screening was similar in non-smokers, former smokers and current smokers: 12.3 ± 4.3 WG, 12.0 ± 4.3 WG, and 12.7 ± 4.5 WG, respectively (*p* = 0.15). Gestational age at OGTT was highest in smokers: non-smokers 27.7 ± 3.5 WG, former smokers 27.3 ± 3.3 WG, and smokers 28.2 ± 3.8 WG, *p* < 0.0001. WG: weeks of gestation. *: vs. non-smokers, symbol inserted only if significant (*p* < 0.05) after Dunnett adjustment for multiplicity.

**Figure 2:**
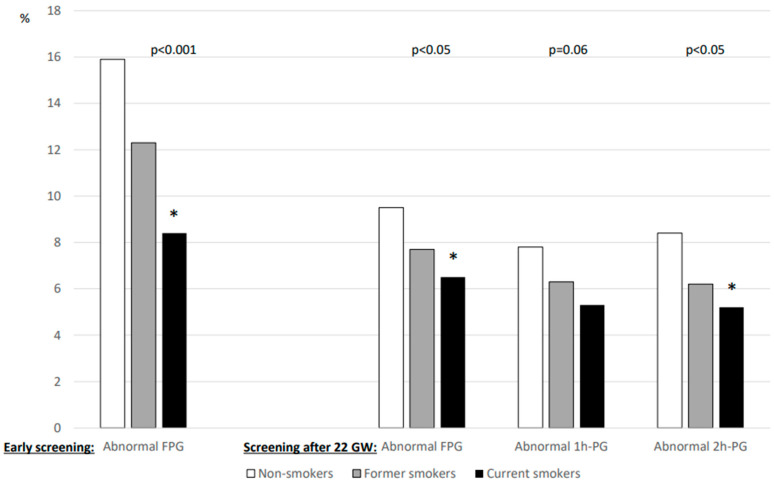
Percentage of abnormal plasma glucose values during screening for hyperglycaemia in pregnancy according to smoking status. Left panel: early screening (before 22 weeks of gestation (WG)), only fasting plasma glucose (FPG) value was measured (n = 5495). Right panel: after 22 WG, a 75 g oral glucose tolerance test (OGTT) was performed with measurement of FPG (n = 9588) and plasma glucose 1 h (1h-PG, n = 8716) and 2 h (2h-PG, n = 8879) after OGTT. WG: weeks of gestation. *: vs. non-smokers, symbol inserted only if significant (*p* < 0.05) after Dunnett adjustment for multiplicity.

The results of glucose values from HIP screening are shown in Figure 1. FPG levels during early pregnancy were lower in current smokers than in non-smokers, whereas those after 22 GW were similar for all three smoking categories. In contrast, the 1h-PG and 2h-PG levels were lower for current smokers screened after 22 WG than for non-smokers. Figure 2 shows that the rates of abnormal plasma glucose values in persons screened for HIP differed according to smoking status.

In the screened population, 19.9%, 15.4%, and 12.3% of non-smokers, former smokers, and current smokers, respectively, tested positive for HIP (*p* < 0.0001) (Table 1). In the multivariable analysis (Table 2), after adjusting for age, BMI, a family history of diabetes, a history of HIP, a history of macrosomic child, and ethnicity, current smokers (odds ratio 0.79 [95% confidence interval 0.64–0.98], *p* < 0.05), but not former smokers (odds ratio 1.02 [95% confidence interval 0.79–1.31]), were still less likely to have HIP than non-smokers.

**Table 2:** Parameters associated with hyperglycaemia in pregnancy in multivariable analyses.

	Odds Ratio	95% Confidence Interval		*p*-Value
		Minimum	Maximum	
Age (for 1 year)	1.061	1.052	1.070	<0.001
Pre-pregnancy body mass index (for 1 kg/m^2^)	1.090	1.080	1.099	<0.001
Family history of diabetes	1.388	1.256	1.533	<0.001
Previous pregnancy history				
History of hyperglycaemia in pregnancy	4.264	3.628	5.011	<0.001
History of macrosomic child	1.476	1.179	1.847	<0.001
Ethnicity				
European	REF			
North African	1.344	1.184	1.526	<0.001
Sub-Saharan African	0.893	0.769	1.036	0.136
Caribbean	0.746	0.589	0.944	0.015
Indian–Pakistan–Sri Lankan	2.767	2.360	3.245	<0.001
Other	1.388	1.145	1.682	<0.001
Smoking status				
Non-smoker	REF			
Former smoker	1.017	0.792	1.306	0.894
Current smoker	0.790	0.636	0.981	0.033

REF: reference.

### 3.3. Possible Inclusion Bias

The lower prevalence of HIP in current smokers may have been due to inclusion bias. As they were less likely to screen for HIP, there may have been a high percentage of undiagnosed HIP in this sub-population [11,12,13]. To explore this possibility, we selected the 1234 current smokers in the study sample and compared (i) the prevalence of risk factors for HIP and (ii) the incidence of adverse HIP-related pregnancy outcomes between the screened (n = 934) and unscreened groups (n = 300). Unscreened women were younger, had a higher parity, were less likely to be employed, and less likely to be of European origin (Table 3).

**Table 3:** Risk factors for HIP according to HIP screening status in the 1234 women who smoked during pregnancy.

	Available Data	Screening		Total	*p*-Value
		No n = 300	Yes n = 934		
Metabolic characteristics					
Age (years)	n = 1234	27.3 ± 6.0	29.2 ± 6.0	28.7 ± 6.1	<0.0001
Pre-pregnancy body mass index (kg/m^2^)		23.2 ± 4.6	23.7 ± 4.8	23.6 ± 4.7	0.1510
Pre-pregnancy obesity		26 (10.1%)	116 (12.7%)	142 (12.1%)	0.2616
Family history of diabetes	n = 1234	75 (25.0%)	271 (29.0%)	346 (28.0%)	0.1780
Employment during pregnancy	n = 1234	71 (24.1%)	421 (45.7%)	492 (40.5%)	<0.0001
Parity	n = 1234	2.3 ± 1.5	2.0 ± 1.1	2.1 ± 1.2	<0.0001
Ethnicity	n = 1234				<0.0001
Sub-Saharan African		16 (5.3%)	56 (6.0%)	72 (5.9%)	
North African		49 (16.3%)	146 (15.7%)	195 (15.9%)	
Caribbean		11 (3.7%)	35 (3.8%)	46 (3.7%)	
European		158 (52.7%)	587 (63.1%)	745 (60.6%)	
Indian–Pakistan–Sri Lankan		1 (0.3%)	1 (0.1%)	2 (0.2%)	
Other		65 (21.7%)	105 (11.3%)	170 (13.8%)	
Previous pregnancy history	n = 1234				
History of HIP					0.2052
First child/parity = 0		104 (34.7%)	406 (43.5%)	510 (41.3%)	
No		190 (63.3%)	500 (53.5%)	690 (55.9%)	
Yes		6 (2.0%)	28 (3.0%)	34 (2.8%)	
History of macrosomic child					0.6541
First child/parity = 0		104 (34.7%)	406 (43.5%)	510 (41.3%)	
No		190 (63.3%)	515 (55.1%)	705 (57.1%)	
Yes		6 (2.0%)	13 (1.4%)	19 (1.5%)	

Data are shown as n (%) or mean (standard deviation). HIP: hyperglycaemia in pregnancy.

Furthermore, unscreened current smokers were more likely to consume recreational substance during pregnancy and had lower GWG (Table 4). They were less likely to have a LGA infant, and their newborns had a lower birthweight. In contrast, they were more likely to experience preterm delivery and perinatal death. In multivariable analysis, after adjustment for age, ethnicity, employment during pregnancy, and parity, not screening for HIP was associated with a lower likelihood of having a LGA infant (odds ratio 0.32 [95% confidence interval 0.12–0.83], *p* < 0.01) and a greater likelihood of preterm delivery (odds ratio 2.14 [95% confidence interval 1.38–3.32], *p* < 0.001) in unscreened current smokers.

**Table 4:** Behaviours during pregnancy and adverse HIP-related pregnancy outcomes, according to HIP screening status in the 1234 women who smoked during pregnancy.

	Available Data	Screening		Total	Odds Ratio	95% Confidence Interval	*p*-Value
		No n = 300	Yes n = 934				
Behaviours during pregnancy	n = 1234						
Alcohol consumption		3 (1.0%)	8 (0.9%)	11 (0.9%)	1.17	[0.31–4.44]	0.7343
Recreation substance consumption		22 (7.3%)	40 (4.3%)	62 (5.0%)	1.77	[1.03–3.03]	0.0353
Maternal outcomes							
Gestational weight gain (kg)	n = 1047	11.2 ± 5.6	13.0 ± 6.2	12.6 ± 6.1			0.0002
Caesarean section	n = 1234	50 (16.7%)	184 (19.7%)	234 (19.0%)	0.82	[0.58–1.15]	0.2436
Hypertensive disorders during pregnancy	n = 1234	10 (3.3%)	38 (4.1%)	48 (3.9%)	0.81	[0.40–1.65]	0.5667
Neonatal outcomes							
Birthweight (g)	n = 1234	2959 ± 564	3117 ± 516	3079 ± 532			<0.0001
Large-for-gestational-age infant	n = 1234	4 (1.3%)	43 (4.6%)	47 (3.8%)	0.28	[0.10–0.79]	0.01
Small-for-gestational-age infant	n = 1234	45 (15.0%)	146 (15.6%)	191 (15.5%)	0.95	[0.66–1.37]	0.7924
Gestational age at birth (WG)	n = 1234	38.8 ± 2.6	39.4 ± 1.7	39.29 ± 1.98			<0.0001
Preterm delivery	n = 1234	41 (13.7%)	64 (6.9%)	105 (8.5%)	2.15	[1.41–3.26]	0.0002
Neonatal hypoglycemia	n = 1234	3 (1.9%)	6 (1.0%)	9 (1.2%)	1.90	[0.47–7.68]	0.4062
Perinatal death and stillbirth	n = 1234	7 (2.3%)	4 (0.4%)	11 (0.9%)	5.55	[1.61–19.11]	0.0062
Birth malformation	n = 1234	2 (0.7%)	11 (1.2%)	13 (1.1%)	0.56	[0.12–2.56]	0.7451

Data are shown as n (%) or mean (standard deviation). HIP: hyperglycaemia in pregnancy, WG: weeks of gestation.

## 4. Discussion

### 4.1. Main Results

Our results show that screened current, but not former, smokers were less likely to have HIP, independently of confounders. Furthermore, their glucose levels during the OGTT were lower than those of non-smokers. We also observed that current smokers were less likely to attend HIP screening. Selection bias did not seem to play any role in this result, as unscreened current smokers were not more likely to have HIP-related risk factors or an LGA infant than their screened counterparts.

### 4.2. Continued Smoking during Pregnancy

Approximately 8% of pregnant women in our series continued to smoke during pregnancy. This percentage is lower than 1986–1988 data for France [17] and lower than 2002–2010 data we collected for women who delivered in our hospital [18]. In contrast, it reflects 2000–2010 data for the United States [19].

### 4.3. HIP Prevalence and Smoking during Pregnancy

While meta-analyses globally show no association between smoking during pregnancy and HIP [8,9], several studies conducted worldwide—including a previous study we performed in our hospital for the 2002–2010 period—highlighted that smoking was associated with less HIP [3,4,18,20,21,22], which reflects our present findings. The odds ratio in those studies ranged from 0.47 [95% confidence interval 0.23–0.96] [22] to 0.90 [95% confidence interval 0.81–1.00] [21]. The odds ratio in the present study fell between these two limits (i.e., 0.79 [95% confidence interval 0.64–0.98]).

The association between smoking and HIP prevalence may differ (i.e., negative, neutral or positive association) because of divergences in how smoking status is assessed, the HIP diagnosis criteria used, and ethnicity-based or between-country differences in other lifestyle behaviours [8,9]. It may also differ because of a divergence in the prevalence of risk factors in HIP smokers and non-smokers. Despite this, results in all [3,4,18,20,22] but one [21] of the aforementioned studies were adjusted for several confounders. Importantly, we did not find any association between former smoking and HIP, despite the fact that smoking cessation may increase appetite and weight gain [23], two potential drivers of HIP. Outside pregnancy, smoking cessation was reported to initially increase diabetes risk [24].

### 4.4. Smoking and OGTT Results

FPG levels during early screening for HIP, and 1h-PG and 2h-PG levels during OGTT after 22 WG, were lower in current smokers than in non-smokers in our study sample. This explains the lower prevalence of HIP in the former group. In women with HIP, defined according to the Carpenter–Coustan criteria, Aulinas et al. also found higher plasma glucose levels at 1 h and lower levels at 3 h after 100 g OGTT in smokers [25]. Elsewhere, Konstantakou et al. found similar patterns in women with and without HIP, specifically higher FPG and lower plasma glucose levels at 3 h after 100 g OGTT in current smokers [26]. These results for current smokers during pregnancy reflect data for current smokers outside pregnancy, specifically lower 2h-PG values than those of non-smokers [27]. Values for former smokers lay between those of current smokers and non-smokers, for both pregnant and non-pregnant populations [26,27]. On the contrary, Zaren et al. reported that 12.4% of mothers who were heavy smokers (i.e., ≥10 cigarettes a day) had a 2h-PG value > 8.5 mmol/L (i.e., the definition of HIP in that study), compared with 9.2% for light smokers and 6.0% for non-smokers [28]. However, in their study, the OGTT was performed late during pregnancy, at 37 WG, which could explain these different OGTT patterns [28].

### 4.5. Why Might Smoking Be Negatively Associated with HIP?

The reason why current smoking may influence OGTT results and ‘protect’ against HIP is still unclear. First, smoking is known to increase insulin resistance and therefore hepatic glucose production [6]. This may increase FPG values in some women [26,27]. However, there is no specific data about the role of smoking in insulin resistance during pregnancy. Furthermore, if smoking actually increases insulin resistance during pregnancy, such an increase might be of minor significance compared with pregnancy-induced insulin resistance [29]. Second, smokers appear to have accelerated gastric emptying during OGTT, leading to earlier glucose absorption, and lower plasma glucose values after OGTT [27]. Third, nicotine-induced insulin release might be involved [30]. Finally, current smokers may have fewer HIP risk factors than non-smokers in general. However, our results were similar after adjusting for most risk factors (we did not have data on BMI at screening time).

### 4.6. Could the Lower Prevalence of HIP in Smokers Be Explained by Less HIP Screening Uptake?

The current smokers in our study sample were half as likely to screen for HIP compared with non-smokers. This reflects previous findings highlighting that current smokers are less likely to undertake preventive screening, irrespective of the disease [11,12,13].

We hypothesized that the rate of HIP we found in current smokers might have been higher if more of this sub-population in our sample had decided to undertake screening. Having said that, unscreened current smokers did not have a higher risk of HIP than their screened counterparts. Specifically, they were less likely to have certain HIP risk factors, such as younger age, but more likely to have a higher prevalence of non-European ethnicity (reflecting previous findings [18]) and a higher parity. Furthermore, given the higher percentages of persons who were unemployed and individuals of non-European origin in this group, they were likely to have greater psychosocial vulnerability (as found elsewhere [3]).

Furthermore, screened current smokers were less likely to have an LGA infant; had the opposite been true, it could have been an indicator of undiagnosed (and therefore untreated) HIP. Psychosocial vulnerability did not appear to be a driver of LGA infants in our sample [16]. Finally, unscreened current smokers were at a higher risk of experiencing preterm delivery and perinatal death than their screened counterparts. This highlights the importance of targeted care during pregnancy for this sub-population.

### 4.7. Strengths and Limitations

Our study has important strengths. First, it involved a large multi-ethnic prospective cohort recruited over a decade. Second, it confirmed results in a similar study of women who delivered at our hospital in 2002–2010 [18]. Third, there was a high rate of HIP screening, we adjusted for several confounders, and we considered former and current smokers.

This study also has limitations. First, weight gain until HIP screening was unavailable. Second, data collected on smoking, alcohol, and recreational substances consumption were self-reported; having said that, previous studies found good validity of self-reported tobacco use when compared with measured plasma cotinine levels [31]. Third, we did not formally assess insulin resistance in the women of our series. Finally, we were not able to evaluate the impact of smoking at different gestational time points and we had no quantitative data on cigarette smoking.

### 4.8. Perspectives

Despite confirming data from a previous study in our hospital [18], the lower prevalence of HIP we found in current smokers in our present study was unexpected. Of course, this result does not advocate smoking to prevent HIP. The higher risk of adverse pregnancy outcomes in current smokers during both pregnancy [32] and the postpartum period [33] neutralizes any apparent protective effect. It is essential to promote smoking cessation during pregnancy.

We plan to complement the work described in the present study with the analyses of the rates of different adverse pregnancy outcomes according to smoking status and HIP status. Smoking may act through nicotine exposure [34]; and altered endometrial maturation [35], immune response [36], and endothelial function [8]. The current study’s results already indicate the need for targeted care for current smokers who do not screen for HIP, given the higher risk of preterm delivery and perinatal death than in their screened counterparts.

## 5. Conclusions

The present study confirmed our previous findings [18] of lower HIP prevalence in current smokers compared with non-smokers, even after adjustment for confounders. Current smokers were less likely to screen for HIP, and selection bias was excluded as a possible reason for this. The pathophysiology explaining this lower prevalence of HIP in current smokers is still unclear and warrants further research.

## Data Availability

The data presented in this study are available on request from the corresponding author due to ongoing studies.

## Supplementary Materials

The following supporting information can be downloaded at: https://www.mdpi.com/article/10.3390/jcm13175149/s1, Figure S1: Flow chart of this study.
