# Supplementary figures and images for: Hyperglycaemia in Pregnancy Is Less Frequent in Smokers: A French Observational Study of 15,801 Women
Source: J Clin Med. 2024 Aug 30;13(17):5149. doi: 10.3390/jcm13175149 (PMC11396654; doi:10.3390/jcm13175149)

**Figure S1: Flow chart of the study**

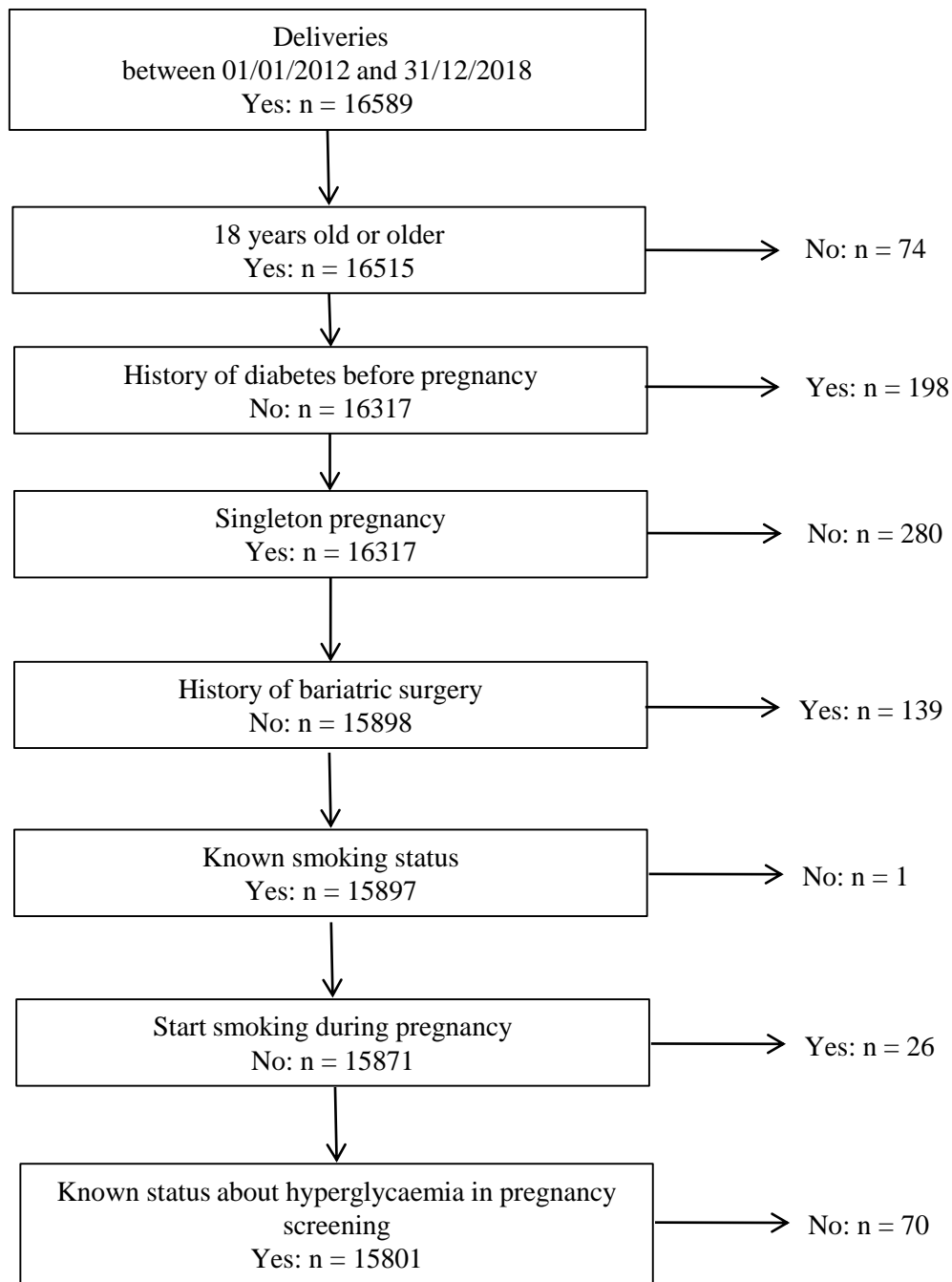

Supplement: Supplementary file 1 [file jcm-13-05149-s001.zip › jcm-3150446-supplementary.pdf]
